# Effects of Blue Light on Dynamic Vision

**DOI:** 10.3389/fpsyg.2019.00497

**Published:** 2019-03-19

**Authors:** Hung-Wen Chen, Su-Ling Yeh

**Affiliations:** ^1^Department of Psychology, National Taiwan University, Taipei, Taiwan; ^2^Graduate Institute of Brain and Mind Sciences, National Taiwan University, Taipei, Taiwan; ^3^Neurobiology and Cognitive Science Center, National Taiwan University, Taipei, Taiwan; ^4^Center for Artificial Intelligence and Advanced Robotics, National Taiwan University, Taipei, Taiwan

**Keywords:** blue light, ipRGCs, dynamic vision, eye pursuit, dynamic visual acuity, kinetic visual acuity

## Abstract

Dynamic vision is crucial to not only animals’ hunting behaviors but also human activities, and yet little is known about how to enhance it, except for extensive trainings like athletics do. Exposure to blue light has been shown to enhance human alertness (Chellappa et al., 2011), perhaps through intrinsically photosensitive retinal ganglion cells (ipRGCs), which are sensitive to motion perception as revealed by animal studies. However, it remains unknown whether blue light can enhance human dynamic vision, a motion-related ability. We conducted five experiments under blue or orange light to test three important components of dynamic vision: eye pursuit accuracy (EPA, Experiment 1), kinetic visual acuity (KVA, Experiment 1 and 2), and dynamic visual acuity (DVA, Experiment 3–5). EPA was measured by the distance between the position of the fixation and the position of the target when participants tracked a target dot. In the KVA task, participants reported three central target numbers (randomly chosen from 0 to 9) moving toward participants in the depth plane, with speed threshold calculated by a staircase procedure. In the DVA task, three numbers were presented along the meridian line on the same depth plane, with motion direction (Experiment 3) and difficulty level (Experiment 4) manipulated, and a blue light filter lens was used to test the ipRGCs contribution (Experiment 5). Results showed that blue light enhanced EPA and DVA, but reduced KVA. Further, DVA enhancement was modulated by difficulty level: blue light enhancement effect was found only with hard task in the downward motion in Experiment 3 and with the low contrast target in Experiment 4. However, this blue light enhancement effect was not caused by mechanism of the ipRGCs, at least not in the range we tested. In this first study demonstrating the relationship between different components of dynamic vision and blue light, our findings that DVA can be enhanced under blue light with hard but not easy task indicate that blue light can enhance dynamic visual discrimination when needed.

## Introduction

Perceiving and analyzing moving objects in order to act immediately and appropriately is essential for survival, and this is true for animals as well as human beings from ancient times to nowadays. For animals and humans in ancient times, they chased preys and hid from predators by the ability called *dynamic vision*—vision for dynamic (constantly changing) stimuli—which is a matter of life and death. For us who have been safe from jungles in modern societies, we turn to use dynamic vision to perform better on sports or video games like shooting and car racing. Thus, another name has been given to this ability—*sports vision*, which includes not only dynamic vision but also hand-eye coordination. In addition, eye movements also play an important role on dynamic vision: when swatting a flying mosquito or a running cockroach, tracking the path of the insect is crucial, or else it will be missed.

Experts from different fields, such as ophthalmologists, vision scientists, and exercise scientists, usually focus on different aspects of dynamic vision. However, since it is hard to draw a clear line between these aspects, we focus on three key abilities critical for dynamic vision: eye pursuit, kinetic visual acuity, and dynamic visual acuity. Continuously and smoothly pursuing the target with eye movements is important for tracking objects. At the same time, since objects are stereoscopic in real world, the ability to perceive motion in depth is also quite often used. One part of dynamic vision called kinetic visual acuity (KVA) is such an ability to analyze the objects moving forward and backward with respect to the horopter (i.e., the fixation plane). In addition, dynamic visual acuity (DVA) is the ability to perceive objects moving leftward, rightward, upward, or downward on the same fronto-parallel plane. Indeed, different brain regions were activated when participants conducted one of these tasks. For example, oculomotor nucleus in the midbrain associates with eye movements, medial superior temporal (MST) associates with expansion targets (Tanaka and Saito, 1989) similar to KVA, and middle temporal visual area (MT/V5) associates with motion perception (Dubner and Zeki, 1971) similar to DVA. Distinct mechanisms seem necessary for these different abilities essential for dynamic vision.

As dynamic vision is so important for both survival and athletic activities, the question on how to enhance human dynamic vision becomes a hot issue, especially in sports/games which usually involve highly competitive situations and varieties of rewards. Human dynamic vision based on motion contrast reaches adult performance at the age of 15 (Schrauf et al., 1999). After reaching the peak, people usually improve their dynamic vision by practicing body-eye coordination reaction time, choice reaction time, and functional field (Ciuffreda, 2011; Schwab and Memmert, 2012). However, some researchers criticized and doubted the evidences of enhancing dynamic vision through training (Barrett, 2009; Lee et al., 2016). Aside from behavioral training, biological stimulation is another approach along with the advancement of technology. For example, Zito et al. (2015) tested the newly developed technique called the high definition transcranial direct current stimulation (HD-tDCS) to replace the sponge electrodes in conventional tDCS, and discovered improvement in motion perception after cathodal HD- tDCS. Still, there are not many studies done on this topic, and not yet did a persuasive approach to enhancing dynamic vision come up.

Previous studies have shown that blue light can enhance human cognition such as alertness (Chellappa et al., 2011) and working memory (Vandewalle et al., 2007; Alkozei et al., 2016), as well as motion perception of mice (Zhao et al., 2014). Exposure to blue light affects human cognition through changing activations in some brain areas, particularly the prefrontal brain regions that are associated with executive functions (Vandewalle et al., 2013; Alkozei et al., 2016). The biological mechanism behind blue light effect is via recently discovered intrinsically photosensitive retinal ganglion cells (ipRGCs, Berson et al., 2002). ipRGCs respond well to light, especially light with wavelength peaking around 480 nm, but drive slow and sustained light responses (Wong, 2012). Though not acting on image forming functions directly like cones, ipRGCs do help modulating the sensitivity of vision (Brown et al., 2012; Horiguchi et al., 2013; Spitschan et al., 2014), even for blind people (Zaidi et al., 2007; Vandewalle et al., 2013). In animal studies, ipRGCs also affect vision by modulating pupil size (Chen et al., 2011). Moreover, ipRGCs respond to moving stimuli, especially that of slow-to-middle-speed motion with every direction (Zhao et al., 2014).

However, how blue light affects human dynamic vision is still unknown. Based on the known mechanisms of ipRGCs, it is possible that dynamic vision could be enhanced under blue light through the activation of ipRGCs. Long and Garvey (1988) used four color lights (white, blue, yellow, and red) as targets on either a dark or a photopic background to measure dynamic visual acuity, and their results showed that only blue light on dark background enhanced DVA performance. On the other hand, because human vision has poor temporal and spatial resolution under blue light (Cavanagh et al., 1987), perhaps dynamic vision would not be enhanced but impaired instead. Yet, different aspects of dynamic vision might have distinct effects when under the exposure of blue light.

In this study, we examined whether blue light could enhance dynamic vision and what might be the mechanisms behind this enhancement if it exists. In Experiment 1, we started from two critical components of dynamic vision which are both essential abilities to perceive motion in real 3D world—eye movement and KVA, and KVA task was refined to eliminate masking effect in Experiment 2. In Experiment 3, DVA of upward and downward motion was measured to see how blue light affects yet another critical component of dynamic vision. Then, we further tested whether difficulty level of DVA could modulate the blue light effect in Experiment 4. Last, whether ipRGCs contributed to blue-light enhancement of dynamic vision was tested in Experiment 5 by using a blue light filter lens.

## Experiment 1: Kinetic Visual Acuity (KVA) and Eye Pursuit Accuracy (EPA)

*Kinetic visual acuity (KVA)*, the ability to perceive an object moving toward or backward, is relatively important among various aspects of dynamic vision, because the object being tracked is usually changing positions in 3D space in reality. When animals hunt their preys or when an athlete follows a tennis ball, the tracers commonly try to move themselves closer to the target, in order to increase the target’s visual resolution. In the meantime, tracers also need to move their eyes promptly to keep their target in the visual field, requiring the ability called *eye pursuit accuracy (EPA).*

**FIGURE 1:**
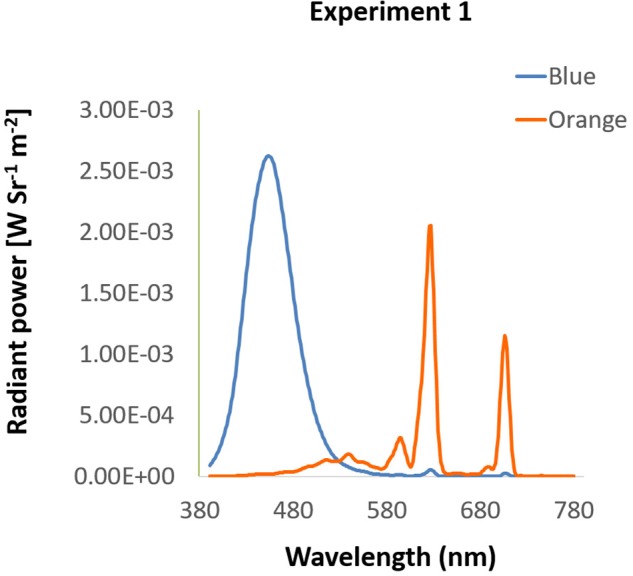
Spectra of background colors in Experiment 1. The spectra of blue and orange background used in Experiment 1 were presented by wavelength (*x*-axis) and radiant power (*y*-axis).

Experiment 1 contained two tasks: the KVA and the EPA task. Each task was measured under two different background colors on the monitor: blue light and control light. Orange was deemed to be the control light in this experiment because it is far away from blue on the color spectrum (see the spectra in Figure 1 and activations of each receptor in Table 2) and yet not biased by its potential stereotypical features. Other colors such as red, serves as a stop or warning signal (Funke, 2010) and increases alertness at night (Figueiro et al., 2009), is likely to carry potential stereotypical features and was thus not used in this study.

### Methods

#### Participants

Sixteen young male adults took part in this experiment (aged from 19 to 35 years, average = 25.9), all with normal or corrected-to-normal binocular vision using the Landolt C vision test. Females—who have menstrual cycles and it has been shown that endocrine system could be affected by ipRGCs activation (Parry et al., 1997; Hanifin and Brainard, 2007) —were not recruited to avoid uncertain variances like short-interval timing (Morita et al., 2005) that might bias the speed judgment. Also, the age was restricted between 18 and 35 years old to prevent visual immaturity or degeneration. To ensure that performances of dynamic vision were free from being affected by visual acuity, participants passed the Landolt C vision test (criterion: 20/20) with naked eyes or corrected vision. Participants signed the written informed consent before the experiment, and were financially reimbursed for their 2-day participation. The study was approved by the Research Ethics Committee at the National Taiwan University (REC code: 201505HS071). All participants gave written informed consent in accordance with the Declaration of Helsinki.

#### Apparatus

Participants were seated in a dimly lit room with their head placed on a chin rest 60 cm from the i-TECH 20″ CRT monitor, in front of which was the EYELINK 2000 eye tracker (SR Research, Mississauga, Ontario, Canada) with 1000 Hz resolution. All stimuli in Experiment 1 were presented on the screen with spatial resolution of 1024 × 768 pixels at 100 Hz refresh rate. Background colors were either blue (luminance: 12.00 cd/m^2^, CIE: 0.1463, 0.0695) or orange (luminance: 14.70 cd/m^2^, CIE: 0.5843, 0.3703), measured by Photo Research Inc’s PR655 (Figure 1). From participants’ view, every single pixel was about 0.037°. All programs in this study were written and presented with MATLAB 8.1 version (The MathWorks) and psychtoolbox 3 versions.

##### Kinetic visual acuity (KVA)

**FIGURE 2:**
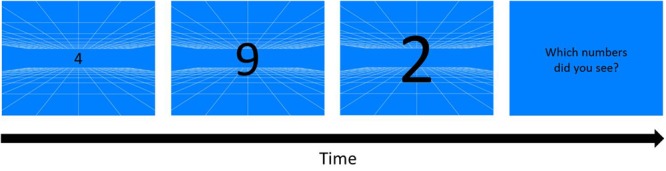
Kinetic Visual Acuity (KVA) Procedure (an example of blue background). A pattern of white grid was drawn on a blue background as a depth cue for participants to build a 12 m^3^ virtual space behind the screen. Whenever participants pressed the space/start key, three randomly chosen one-digit numbers would appear from the middle of the display, and enlarge and sequentially change the numbers in cycles. As the numbers disappeared, three numbers reported by participants were typed by the experimenter.

A blue or orange background with white grid (0.037° of each line) served as depth cue (Figure 2) was presented on the monitor, creating a virtual space of 12 m^3^ behind the screen by means of projection matrix in OpenGL in MATLAB (OpenGL Wiki, 2018). Three black randomly chosen numbers (0–9) appeared when participants pressed the space bar, and the numbers were enlarged from about 0.91° to 18.43°of visual angle, simulating a 20 cm high object that initiated from 12 m away behind the screen and moved toward the participants. In each trial, three moving and enlarging numbers each sequentially changed to its next number in 5 cycle with the fixed order every 80 cm in the simulated space.

A one-up one-down staircase procedure was used to measure the KVA threshold: moving numbers speeded up when participants reported the numbers correctly no matter what the sequence was, and speeded down when they reported any numbers incorrectly. Note that the word “threshold” used here indicated participants’ abilities of dynamic vision: higher speed threshold represents better dynamic vision performance. Though using the one-up one-down staircase procedure, the chance level was very low (8%) because participants had to discriminate three numbers out of ten (0–9) in each trial. All speed in KVA tasks were calculated from the distance in the virtual space because the real visual angle varied every frame and was hard to be revised to a linear function. The initial speed was 8 m/s, and the step size was 0.5 m/s. There were eight reversals in each staircase, and participants finished six staircases. Only the average of the last six reversals in each staircase and the last five staircases were included in the calculation of the speed threshold (Levitt, 1971).

##### Eye pursuit accuracy (EPA)

**FIGURE 3:**
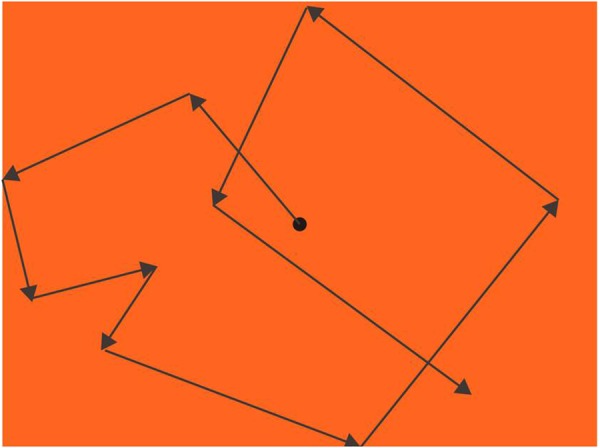
Procedure of eye pursuit accuracy (EPA; an example of an orange background). A black dot moved with a changing speed from slow to fast, increasing its speed every sec and randomly changing its speed every 0.3∼2 sec or when hit the boundaries. Participants’ task was to keep their fovea on the dot as precisely as they could, and their eye movements were recorded by EYELINK 2000.

A black dot with 0.74° diameter was moving from the center after participants pressed the start key. The moving dot started at the speed of 7.40°/s; for the first 20 s, the speed increased 3.70°/s every second, and then increased 1.85°/s every second after that. The dot always went straight, changing its direction randomly every 0.3∼2 s (jittered, Nishitani et al., 1999) or when the dot hit the boundaries of the screen (Figure 3).

To see how participants pursued the dot, we calculated, for each frame, the average overall, *x*-axis-only, and *y*-axis-only shortest distance between the target dot and the eye position. We separated *x*-axis-only and *y*-axis-only distances from the overall distance because horizontal and vertical eye pursuit may have different underlying mechanisms (Rottach et al., 1996).

#### Procedure

Participants were randomly assigned to do the experiments in one of the two counterbalanced conditions: background (blue/orange) and task orders (KVA/EPA). Since we aimed to test how different background colors affect dynamic vision, participants conducted the experiment under blue/orange light on the first day and the other background color on the next day. This manipulation was to make sure that the results were not due to practice effect—better performance on the second day. Moreover, we were also interested in participants’ general dynamic vision ability, by analyzing the correlation between KVA and EPA tasks, the order of the two tasks was also counterbalanced across participants.

After tested their left-eye, right-eye, and binocular visual acuity by the Landolt C test, participants were seated in a dimly lit room for a 5-min light adaptation. It took 5 min to adapt to the same background colors as when with task, in order to activate slow-adapted ipRGCs (Wong, 2012). During adaptation, participants orally answered a questionnaire about their exercise and video game playing habits while opening their eyes toward the screen. Afterward, participants began the experiment from either KVA or EPA.

In the KVA session, participants saw the grid on the background color, and then they were asked to press the space bar to start. After each trial, the experimenter was told what numbers participants saw and typed them for the participants, because the number keys on the keyboard were not separated from other irrelevant keys, which might lead participants to press the wrong key and be distracted by key pressing.

In the EPA session, nine-point calibration and validation procedure were administered before starting the EPA experiment. Participants’ left eye was tracked by the eye tracker with a sampling rate of 1000 Hz, and they were asked to stare at the central dot when pressing the space bar to start the trial. Before each trial, the experimenter checked the status of eye tracker to make certain that participants’ eye movements were recorded precisely. Participants were instructed to pursue the moving dot by fixating it as possible as they could, without using the corners of their eyes to pursue. Three 45-sec pursuit trials were conducted, and participants took a 90 s (or more) self-paced break between two trials.

Two-tailed *t*-test was conducted by SPSS to examine whether performance differed in blue and orange background. We were also interested in the relationship between KVA and EPA, testing whether performances of KVA were correlated with EPA patterns because these two are both critical components of dynamic vision. Bivariate correlation of SPSS was used to compute the Pearson correlation.

### Results

#### Kinetic Visual Acuity (KVA)

**FIGURE 4:**
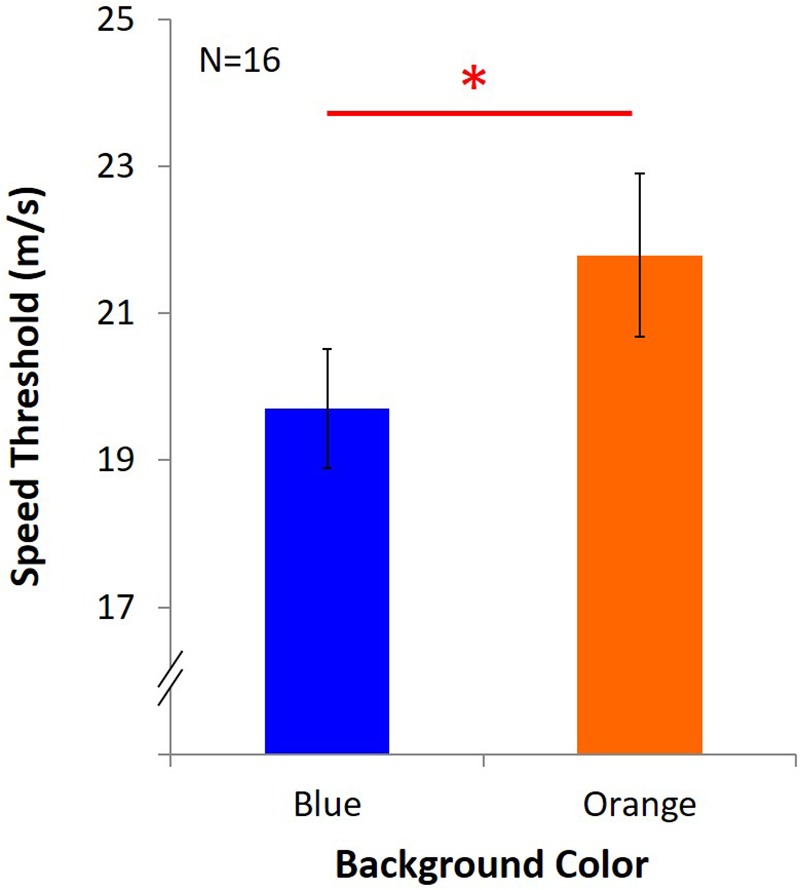
Results of KVA. Two colored bars represent the average speed threshold under blue or orange background. Error bars represent one standard error of the mean (SEM), and the ^∗^statistical significance of the comparison (*p* < 0.05).

Average speed threshold of KVA under blue light was 19.70 m/s ± 3.25, and it was 21.79 m/s ± 4.43 under orange light (Figure 4). The result showed that KVA was significantly better under an orange background [*t*(15) = -2.84, *p* = 0.01, *d_KV A_* = 0.71].

#### Eye Pursuit Accuracy (EPA)

**FIGURE 5:**
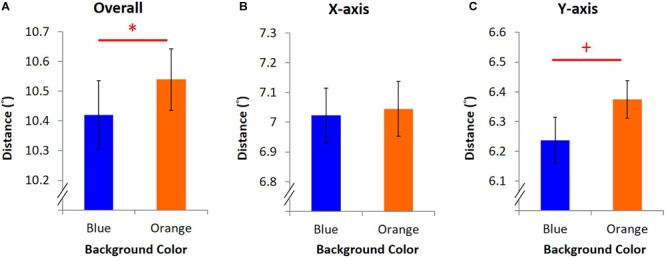
Results of EPA. Different colored bars stand for the average distance under blue/orange light. Error bars represent one SEM. ^∗^Significant result (*p* < 0.05), + marginally significant (*p* < 0.10). **(A)** The average of overall distance was calculated as the mean of the shortest distance between eye position and dot position every 10 ms in three trials. **(B)** *X*-axis-only distance was the average of the shortest horizontal distance between fixation and the dot. **(C)** *Y*-axis-only distance was computed as **(B)** but with the shortest vertical distance.

In the EPA task, average overall distance was 10.42° ± 0.11 under blue light, significantly shorter than 10.53° ± 0.10 under orange light [*t*(15) = -2.24, *p* = 0.04, *d_EPA_* = 0.56, Figure 5A]. A shorter average overall distance implied that EPA was better under blue light. Furthermore, the *y*-axis-only distance was 6.24° ± 0.07 under blue light, marginally shorter than 6.37° ± 0.06 under orange light [*t*(15) = -1.84, *p* = 0.09, Figure 5C]. Nevertheless, on the *x*-axis-only distance, it was 7.02° ± 0.09 under blue light, which was not different from 7.04° ± 0.09 under orange light [*t*(15) = -0.45, *p* = 0.66, Figure 5B].

#### Correlation Between KVA and Eye Pursuit

As Table 1 shows, KVA was negatively correlated with overall (*r* = -0.550, *p* = 0.027) and *y*-axis-only distance (*r* = -0.535, *p* = 0.033), and marginally correlated with *x*-axis-only distance (*r* = -0.465, *p* = 0.069) under blue light. However, there were no correlations for the three situations under orange light.

**Table 1:** Correlation between KVA and EPA.

	Overall	*x*-axis	*y*-axis
Blue light	–0.550^∗^	–0465^+^	–0.535^∗^
Orange light	–0.377	–0.340	–0.296
Average	–0.483^+^	–0.361	–0.574^∗^

*Bivariate correlations were computed using Pearson correlation. ^∗^Significant results (*p* < 0.05), + marginally significant results (*p* < 0.10).*

The performances under blue light and orange light were combined to see the general ability of dynamic vision of each participant. We observed that KVA had significant correlation with *y*-axis-only distance (*r* = -0.574, *p* = 0.020) and marginal correlation with overall distance (*r* = -0.483, *p* = 0.058).

#### Questionnaire

We conducted the same questionnaire of the exercise and video game playing habits of types and frequencies in every experiment of this study. However, no correlation was found, so it would not be mentioned in the following experiments.

### Discussion

We found that KVA performance was better under orange light. On the contrary, EPA performance was better, especially with shorter *y*-axis distances, under blue light. Furthermore, KVA performances were related to distance of EPAs with respect to the target dot: The better the KVA, the shorter distance between participants’ eyes and the target dot.

#### KVA and Critical Flicker Fusion Threshold (CFFT)

For KVA, speeding up stimuli required higher temporal resolution, which was related to the critical flicker fusion threshold (CFFT). Although CFFT was not measured here, in this present experiment, three numbers repetitively appeared at the same position on the screen, which was similar to flickers in that each number only appeared briefly and was then quickly replaced by the next number. It thus required better temporal resolution similar to CFFT to accomplish the task, and the result was also in the same direction as the CFFT results indicated: worse under blue light (Brindley et al., 1966). Perhaps, it was not “enhanced” KVA performance under orange light, but “impaired” KVA performance under blue light due to poorer CFFT.

#### Eye Pursuit Accuracy (EPA)

Results of EPA under blue light showed shorter overall and *y*-axis-only distances, but no difference in the *x*-axis-only distance. Rottach et al. (1996) tested the horizontal and vertical eye pursuit, and the authors discovered that horizontal pursuit was better when predictable waveforms were used, while vertical pursuit had greater eye accelerations when initiation of pursuit was tested. Since the dot in this present experiment moved randomly like the initiation condition in Rottach et al., it showed a similar pattern that the target-eye distance was shorter in *y*-axis than in *x*-axis, suggesting that horizontal and vertical eye pursuit were separately controlled.

#### KVA and EPA

The correlation between KVA and EPA as shown in table 1 was similar between (1) the blue background and (2) general dynamic visual ability when results under blue and orange background were combined (i.e., “average” in Table 1). Yonehara et al. (2009) found that cells in the medial terminal nucleus, the principal nucleus of the accessory optic system receiving signals from direction-selective ganglion cells in the retina coding direction of image motion, only were activated by optokinetic stimuli (crucial for eye pursuit) of vertical motion. This may be the reason why vertical pursuit was found more prominently associated with KVA here.

## Experiment 2: KVA Without Masking

The stimuli of the KVA task used in Experiment 1 were presented one after another in a short time interval, inducing a masking effect that one number might have masked another. Since human’s temporal resolution was poor under blue light (Cavanagh et al., 1987), perhaps it triggered a greater masking effect under blue light than under orange light, leading to the results of better KVA under orange light in Experiment 1. Hence, in this experiment, we aimed to measure the KVA threshold without numbers being masked. The purpose of this experiment was to clarify that better KVA performance under orange light was not just a masking effect—an effect that was proven to be impaired under blue light—but due to KVA itself. To eliminate the spatial masking effect, we separated the three target numbers into three different positions (up, left, right) on a same object – a moving cube. The virtual space was created by the white grid by means of OpenGL, and the presentation time of each number was the same as that in Experiment 1. In this way, we controlled the presentation time of targets and the task (discriminating and memorizing three numbers), but isolated three numbers in three positions to get rid of the potential masking effect. In Experiment 2, we only conducted the modified KVA task.

### Methods

#### Participants

Another group of 16 male participants with ages between 19 and 32 (average = 23.9 years) were recruited, all with normal or corrected-to-normal binocular vision, and signed informed consent before the experiment.

#### Apparatus

**FIGURE 6:**
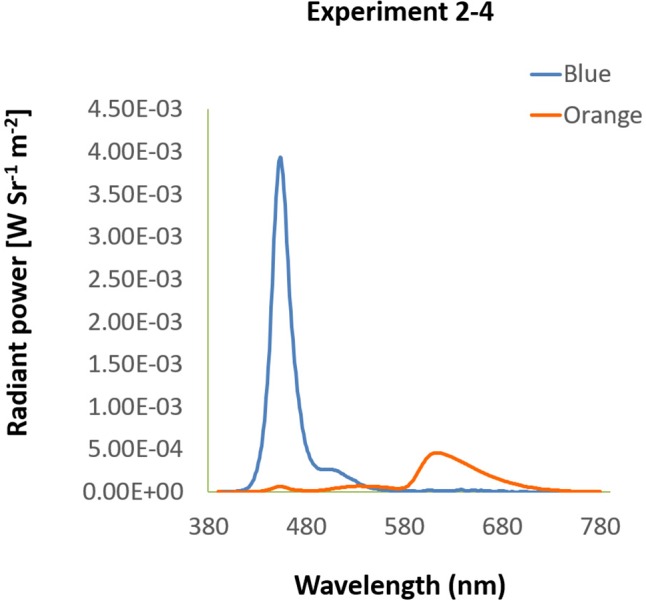
Spectra of background in Experiment 2–4. The spectra of blue and orange background used in Experiment 2–4 were illustrated by wavelength (*x*-axis) and radiant power (*y*-axis).

Participants were seated in a dimly lit room with their head on a chin rest at a distance of 60 cm from a 27″ LED screen (EIZO FORIS FS2735 QHD FreeSync), also used in the following experiments. Background were either blue (luminance: 8.51 cd/m^2^, CIE: 0.1454, 0.0544) or orange (luminance: 9.34 cd/m^2^, CIE: 0.6173, 0.3605), measured by Photo Research Inc’s PR 655. Their spectra are shown in Figure 6. All stimuli were presented on the screen with spatial resolution of 1024 × 768 pixels and a 120 Hz refresh rate. Under this setting, each pixel was about 0.037° at the viewing distance of 60 cm.

#### Stimuli and Design

**FIGURE 7:**
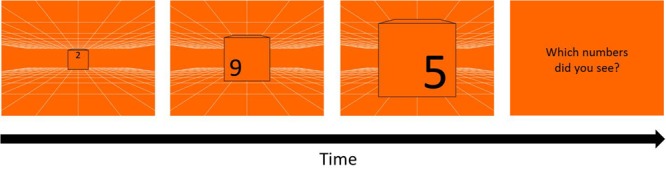
Procedure of KVA without masking effect. Three randomly chosen numbers (13.3 cm height) repeatedly appeared in the order of up-, left-, and right-position for 5 cycles on a 30 cm^3^ cube moving from 12 m toward participants. After each trial, participants had to report the three numbers in the same order as their appearing order (up, left, right). A one up one down adaptive staircase procedure was used to calculate the threshold of KVA: speed up when participants answered correctly and speed down when answered incorrectly, in terms of either the numbers or the order.

The same white grid used in Experiment 1 was presented on either blue or orange background as depth cue and created a virtual space of 12 m^3^ (Figure 7). To avoid the masking effect, three black randomly chosen numbers (0–9) were printed at the up-, left- and right-position on the front of a 30 cm^3^ cube, with 13.3 cm height each, and the cube was enlarged from about 1.36° to 26.57°of visual angle, which looked like a cube moving from 12 m toward participants. In each trial, three numbers on the cube appeared sequentially with the order of up-, left-, and right-position, and changed in 5 cycle with the fixed order every 80 cm in the simulated space, replicating the time intervals as the stimuli in Experiment 1. After a trial, participants reported the three numbers in the same order as the appearing order: up, left, right. The answer was regarded as wrong when the numbers or the order were different from the stimuli.

The threshold was calculated by a one-up one-down adaptive staircase procedure, with 8 reversals in 6 staircases each. Due to the restricted rules in answering three correct numbers and their orders, there were 720 possibilities (10 × 9 × 8) in one trial, equivalent to about 1% chance level, which was impossible to be guessed. The initial speed was 23.26 m/s, and the maximum speed was 116.28 m/s, with the step size of 3.49 m/s before the second reversals, the step size of 1.16 m/s between second and third reversal, and that of 0.58 m/s after the third reversal. The final threshold was the average of last 6 reversals of every staircase excluded the first one.

The same two-tailed paired *t*-test as in Experiment 1 was computed by SPSS to test whether KVA performances were still better under orange light than under blue light.

#### Procedure

Participants were randomly assigned to conduct the experiments under either blue or orange background on the first day. As in Experiment 1, after their monocular and binocular visual acuity tested by the Landolt C task, participants were instructed to be seated in a dimly lit room for a 5-min adaptation. After that, when the white grid pattern appeared on the background, participants were instructed to press the space bar to begin whenever they were ready. At the end of each trial, participants typed the three numbers sequentially by pressing the number keys on the keyboard.

### Results

**FIGURE 8:**
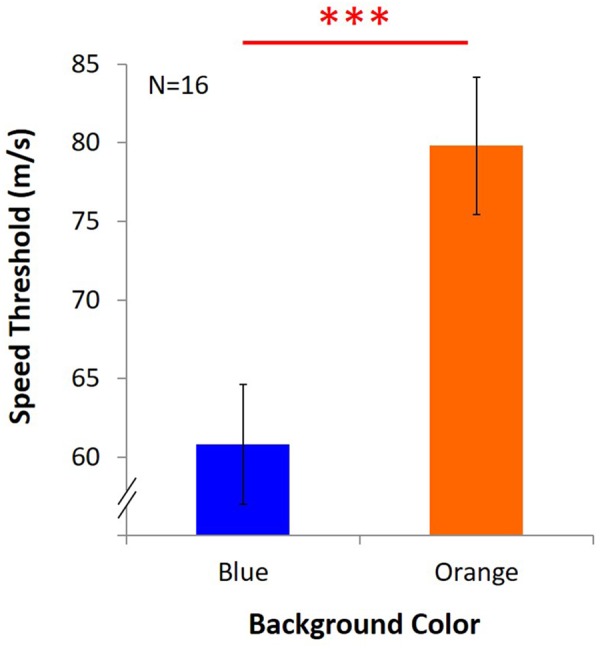
Results of KVA without masking effect. Two bars represent the threshold of KVA under blue and orange light, and *Y* axis indicates the average speed threshold. ^∗∗∗^significant result (*p* < 0.001).

Average speed threshold under blue light was 60.79 m/s ± 3.81, and it was 79.82 m/s ± 4.37 under orange light (Figure 8). The results showed that despite the withdrawal of the masking effect, KVA was still significantly better under the orange background than the blue one [*t*(15) = -6.531, *p* = 0.000, *d_KV Aw/o-masking_* = 1.69].

### Discussion

The results showed the same effect of better KVA performances under orange light than under blue light, like the results in Experiment 1, indicating that indeed KVA impairment under blue light was not due to poor temporal resolution and lower CFFT as demonstrated by Brindley et al. (1966). Therefore, how could blue light affect differently on KVA task and EPA task in Experiment 1? In the KVA task, participants had to do more than just following the target like in the EPA task. Hence, we tested whether the distinctions of the blue light effect on these two tasks were due to the task itself.

## Experiment 3: Dynamic Visual Acuity (DVA)

In Experiment 1, the blue light enhancement effect was found only in the EPA task, but not the KVA task. There was yet another possibility for this result: the presentation of digits in KVA was in the fovea, where ipRGCs and S cones are lacking, which might be the cause for the discrepancy in the results. Therefore, we conducted an experiment similar to KVA but in which the stimuli were presented across different visual fields, so-called dynamic visual acuity (DVA), in Experiment 3.

We followed and modified the paradigms used to measure DVA in previous studies. Ludvigh and Miller (1958) started to use a measure similar to static vision test, which Brown (1972) refined as projecting moving Landolt C on a white screen 1.5 m from observers. AtheleVision, software made by a Japanese vision scientist Hisao Ishigaki, used a ball with numbers printed at different locations of the ball rolling at the center to test DVA of athletics (Liu et al., 2010). We used central moving numbers without the rolling ball and two vertical lines were added instead.

In order to examine participants’ DVA, like measuring KVA in Experiment 1, both motion detection and object discrimination should be tested. In Experiment 3, we examined whether upward-motion and downward-motion DVA could be affected under blue light. On account of finding that blue light affected more on *y*-axis pursuit in Experiment 1, only vertical motion was administered in this experiment.

### Methods

#### Participants

Twenty-four males were recruited (aged from 20 to 34, with average 24.5 years old). Vision, age, and gender of all participants met the same criteria as in Experiment 1. The written informed consent was collected from every participant before the experiment started.

#### Apparatus

The same blue or orange background was displayed on the LED monitor as in Experiment 2, which was at a distance of 80 cm from the participants. All stimuli in this experiment were presented on the screen with spatial resolution of 1920 × 1080 pixels and a120 Hz refresh rate, making each pixel being about 0.02° from participants at a viewing distance of 80 cm under this setting. The same apparatus was used in Experiment 4 and 5.

#### Stimuli and Design

**FIGURE 9:**
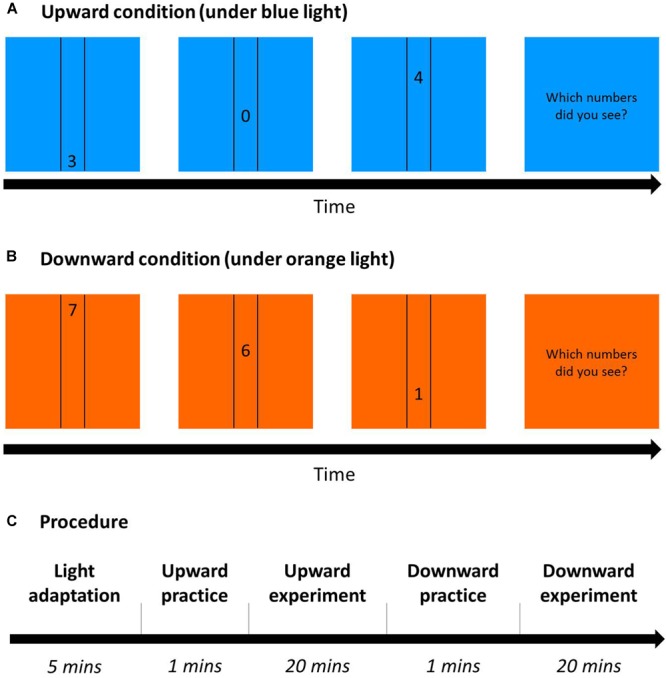
Dynamic visual acuity (DVA) Procedure. **(A)** Upward condition (an example under blue light): 3 randomly chosen numbers sequentially moved between two vertical lines from down to up on a blue square as background. After the trials, participants had to report the three numbers in the same order as they were presented. **(B)** Downward condition (an example under orange light): Similar to **(A)**, but numbers moved from up to down with an orange background. **(C)** The procedure of DVA on each day for 2 days: After 5-min blue/orange light adaptation (the same color as the task), participants practiced correctly for five trials and then conducted the DVA task in either the upward or downward condition (this figure shows the upward condition first), and did the other condition afterward.

A square (22.72° × 22.72°) in blue or orange as background was displayed during the whole experiment. Three randomly chosen numbers (0–9, size 1.89°) moved sequentially in either upward or downward direction at the center of the display, between two vertical black lines at a distance of 4.53° (Figure 9A,B). Only one number appeared on the monitor at a time, changing to the next number when it had moved a distance of 7.57° (1/3 of the monitor). Participants’ task was to report the numbers in the same order as they appeared. A one-up one-down adaptive staircase procedure was used with eight reversals for one staircase and six staircases in each condition. The initial moving speed was 19.80°/s, and the step size was 2.97°/s before the first reversal, 0.99°/s between the first and second reversal, and 0.50°/s after the second reversal.

#### Procedure

A 2 (background: blue/orange) × 2 (direction order: upward/downward) within-subject design was used, and all conditions were randomly assigned across participants. Two-way ANOVA was computed using SPSS, with two factors: light (blue/orange) and direction (upward/downward). Participants underwent the vision test and then were seated in a dimly lit room for light adaptation for 5 min. After the adaptation, participants began the six staircases of upward/downward DVA after practicing, and then finished the other direction of practice trials and formal experiment (Figure 9C). In each trial, the colored background square and the black vertical lines were shown on the monitor, and not until participants pressed the space/start bar would the target numbers appear to move. Then, participants typed the three numbers they saw in the same order as the stimuli appeared. If participants reported correct numbers but with a wrong order, then the answer would be regarded as wrong and the speed of the next trial would be decreased.

### Results

**FIGURE 10:**
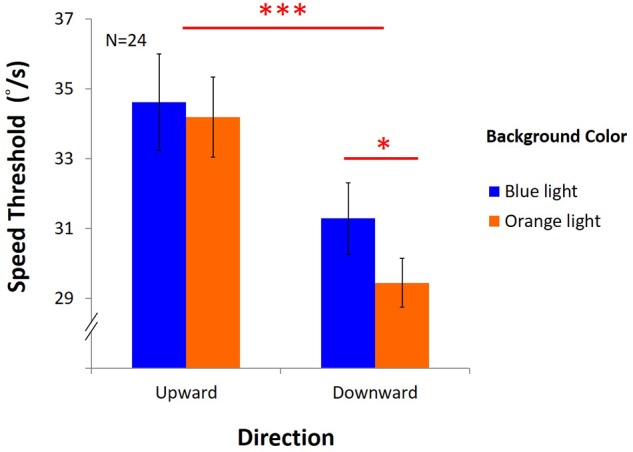
Results of DVA. *X*-axis shows directions of upward and downward condition, and *y*-axis indicates threshold for speed. Blue bars represent the average speed threshold under blue light, and orange bars, average speed threshold under orange light, with SEMs as error bars.

Figure 10 showed that the performance was better under blue light than under orange light [*F*(1,23) = 6.42, *p* = 0.02, η*^2^* = 0.22], and better with upward direction than downward direction, as expected [*F*(1,23) = 33.83, *p* < 0.001, η*^2^* = 0.6]. However, there was a marginal interaction between light and direction [*F*(1,23) = 4.18, *p* = 0.05, η*^2^* = 0.15]. In the upward condition, average DVA speed threshold was 34.12°/s ± 1.38 under blue light, no different from that with 33.87°/s ± 1.15 under orange light. However, in the downward condition, average speed was 30.69°/s ± 1.02 under blue light, significantly higher than 28.96°/s ± 0.70 under orange light (*post hoc*: *p* = 0.004). We found that blue light only enhanced DVA with downward motion, but not with upward motion.

### Discussion

Performance of DVA was better under blue light than under orange light, which was consistent with the result of EPA in Experiment 1. Taken together the results from Experiment 1 and 3, this showed that blue light indeed enhanced dynamic vision when stimuli were not only presented in the fovea. In the KVA task in Experiment 1 and 2, participants had to memorize the three numbers continuously displayed in cycle, requiring more capacity than the EPA task, in which participants merely looked at the moving dot. However, in the DVA task in Experiment 3, participants should memorize three numbers presented like in the KVA task, and still showed the blue light enhancement effect as obtained from the EPA task. Thus, the opposite blue light effects in Experiment 1 (i.e., *enhancement* in EPA and *impairment* in KVA) may not be due to different strategies participants used or differences in memory capacities between EPA and KVA. Rather, it should be due to the difference between foveal and peripheral presentation of the stimuli that affect activations of S cones and/or ipRGCS, or post-receptor processes further downstream that require involvement of foveal or peripheral processing.

Also, our DVA result echoed that in Long and Garvey (1988) that DVA was better under blue light among other colors in the dark-adapted condition. In the current study, the speed threshold in the upward condition was higher than that in the downward condition, which was also discovered in Seya et al. (2015) who used random-dot patterns and found that vection magnitude was larger in upward motion than downward motion.

In summary, higher speed threshold was found under blue light especially with downward motion, indicating better performance with downward motion under blue light. However, we wondered why the blue light enhancement effect occurred only in the downward condition. Since performance in the upward condition was better, we hypothesized that upward DVA was an easier task, compared to downward DVA. Hence, in Experiment 4 we tested whether difficulty level could modulate the blue light enhancement effect.

## Experiment 4: DVA With Difficulty Level Manipulated

In this experiment, we aimed to investigate whether difficulty level indeed affects the enhancing DVA effect under blue light. Previous studies have shown that taking an easy task requires only little capacity (Kahneman, 1973), which may lead to small or no effect on the manipulated factor. We conducted easy and hard task with the same upward direction as in Experiment 3, but changed contrast of the moving numbers to reduce the visibility by lowering the contrast (Robson, 1966). Thus, we decreased the target-background contrast by changing the black numbers to gray, which we called a hard task. The original condition of black numbers was called an easy task.

### Methods

#### Participants

Another group of 16 males were recruited (aged from 19 to 29, with average 22.5 years old). All participants met the same criterion as in Experiment 3.

#### Stimuli and Design

Same stimuli and design in Experiment 3 were used in this experiment: three moving numbers were presented between two vertical lines. However, in this present experiment, only the upward condition was conducted. To increase the difficulty level, we adjusted the luminance of the moving numbers to gray (1.58 cd/m^2^, CIE: 0.3124, 0.3192). The luminance of black numbers was nearly 0. The Michelson contrast (max - min) / (max + min) was 59% for the gray numbers in the hard task and 100% for the black numbers in the easy task.

#### Procedure

Using the same setup as in Experiment 3, the experiment with either blue or orange background was conducted in two separated days of the same time period. The main task (either easy or hard) would take place after a 5-min light adaptation and practice trials. We analyzed a two-way ANOVA with two factors, light (blue/orange) and difficulty (easy/hard).

### Results

**FIGURE 11:**
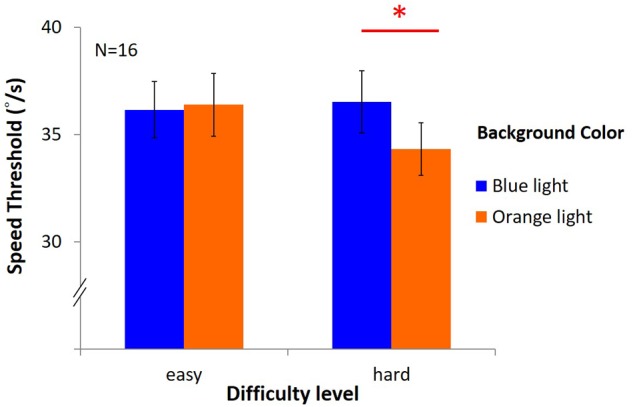
Results of DVA in two difficulty levels. The *x*-axis indicates the difficulty of the task (easy or hard), while *y*-axis depicts the speed of mean reversals in the staircase procedure. Again, colored bars represent background, and the error bars represent SEMs.

The results (Figure 11) showed no significant main effects [light: *F*(1,15) = 1.22, *p* = 0.29, η*^2^* = 0.08; difficulty: *F*(1,15) = 3.27, *p* = 0.09, η*^2^* = 0.18]. However, there was an interaction between light and difficulty [*F*(1,15) = 4.61, *p* = 0.05, η*^2^* = 0.24]. Hence, *post hoc* test (LSD) was estimated as below: in the easy task, the average speed was 36.16°/s ± 1.32 under blue light, not different from 36.39°/s ± 1.46 under orange light; in the hard task, the speed threshold was 36.52°/s ± 1.46 under blue light, significantly higher than 34.33°/s ± 1.23 under orange light[(*post hoc* (LSD): *p* = 0.03].

### Discussion

Dynamic visual acuity can be enhanced under blue light only with hard task but not easy task. It indicates that the lack of blue light enhancement effect in upward motion in Experiment 3 may be due to its easiness, but not due to different mechanisms between upward and downward motion.

Although the manipulation of difficulty level was only marginally significant, there was a possibility that the facilitation in the hard task was so immense that it reached the performance in the easy task. Because in both tasks, participants only conducted the upward condition, not the downward condition like in Experiment 3, it may result in a larger practice effect.

## Experiment 5: DVA With ipRGCs Level Manipulated

In Experiment 3, we found that DVA was better under blue light. In this experiment, we tested whether it was due to the activation of ipRGCs. To examine the effect of ipRGCs, a blue-light filter lens was used to filter out half of the ipRGC activation while keeping the color and luminance the same (Shih et al., 2016).

### Methods

#### Participants

A different group of 16 young male adults were recruited (aged from 19 to 28, with average 22.38 years old). Three participants reported having their left eye as their dominant eye. Participants looked from their own hands holding a circle by either left or right eye to test which was their dominant eye, and used their dominant eye to look through the filter lens.

#### Apparatus

**FIGURE 12:**
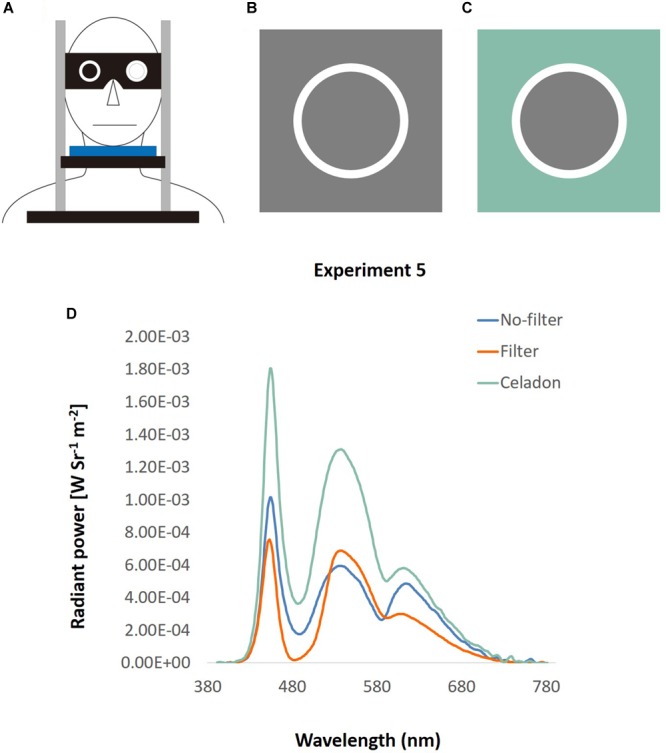
Apparatus of DVA with filter. **(A)** A pair of goggles with two hollows was equipped on a chin rest. Participants looked through the hollow with their dominant eye, and their non-dominant eye was blocked by a black lens. The location of lens was changeable, so that it could fit different conditions and participants’ dominant eye. **(B)** No-filter condition: the square was what displayed on the screen, and the circle was what participants looked directly through the hollow of the goggle. **(C)** Filter condition: the screen displayed a celadon square, and was looked as gray through the filter lens. **(D)** The spectra of the background used in Experiment 5, illustrated by wavelength (*x*-axis) and the radiant power (*y*-axis). The green line represents the color spectrum of the original celadon color, and the blue line indicates that of gray color in the no-filter condition. The orange line depicts the spectrum of gray color looked through filter in the filter condition.

This experiment followed the setting in Experiment 3, but with a pair of adjustable goggles added on the chin rest (Figure 12A). There were two hollows on the goggles that could place two lenses according to different conditions. In the no-filter condition, participants stared at the screen directly through the hollow with their dominant eye, and were blocked with a black filter with their non-dominant eye. In the filter condition, a filter lens, which filtered out the light wavelength from 488 to 514.5 nm, was equipped at participants’ dominant eye to lower the ipRGCs activation level to half of that in the no-filter condition (Shih et al., 2016). To equate the luminance and the color of background in the two conditions, gray (RGB: 128, 128, 128) was used in the no-filter condition (Figure 12B), and celadon (RGB: 136, 188, 170) in the filter condition (Figure 12C). This celadon was determined using the functions in Shih et al. (2016) to let participants look through the filter lens the same as gray. Besides, the luminance was similar in the two conditions (32.01 and 30.63 cd/m^2^, measured by Photo Research Inc’s PR655, Figure 12D).

#### Stimuli and Design

All materials and design were the same as in Experiment 2, except that the background was substituted to gray (no-filter) and celadon (filter). Hence, a 2 (lens: no-filter/filter) × 2 (direction: upward/downward) within-subject design was conducted. Two factors were manipulated in the ANOVA test: lens (no-filter/filter) and direction (upward/downward).

#### Procedure

The procedure was similar to that in Experiment 2, however, we tested the participants’ vision only for their dominant eye. Before going into the dimly lit room, the goggle was already set, so that participants did not know which condition it was. Participants were instructed to sit still and to look out from their dominant eye during adaptation and the whole experiment.

### Results

**FIGURE 13:**
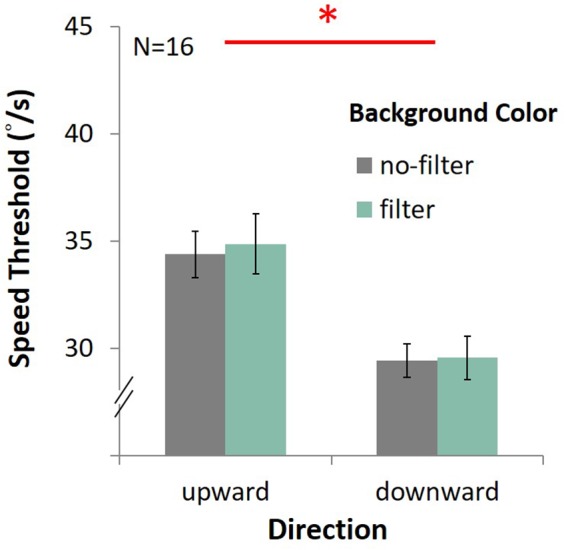
Results of DVA with filter. The four bars indicate the average speed (*y*-axis) of upward or downward motion (*x*-axis) in different background conditions (gray for the no-filter condition, celadon for the filter condition). Error bars are SEMs.

In the upward condition, the average speed was 34.37°/s ± 1.08 under the no-filter condition, and 34.87°/s ± 1.39 under the filter condition (Figure 13). In the downward condition, the average speed was 29.43°/s ± 0.78 with no filter, and 29.55 °/s ± 1.01 with filter. There was only the main effect of direction, showing that performance in upward motion was better than downward motion [*F*(1,15) = 70.27, *p* < 0.001, η*^2^* = 0.82]. No main effect on lens [*F*(1,15) = 0.25, *p* = 0.63, η*^2^* = 0.02] and no interaction between lens and direction [*F*(1,15) = 0.08, *p* = 0.78, η*^2^* = 0.005] were observed.

### Discussion

Again, we found better performance (higher speed threshold) for upward motion than downward motion, a result consistent with that obtained in Experiment 3. Nevertheless, there was no enhancing effect in the no-filter condition, showing that blue light effects on DVA in Experiment 3 and Experiment 4 were probably not due to mechanism of ipRGCs.

Still, there was a possibility that the ipRGCs effect could not be triggered due to the low stimulus intensity. Since ipRGCs respond to light wavelength peaking at 480 nm, which appears blue for human, the gray background in this experiment contained less short-wave light than the blue background in Experiment 3 and 4. Perhaps, in this present experiment, the ipRGCs activation level was not high enough to enhance DVA.

Table 2 shows the activation level for three types of cones and ipRGCs; all stimulations from the experiments were calculated based on 10-deg fundamentals calculated from CIE 2006. In Experiment 1–4, activation of S cones and ipRGCs were much higher under blue background than under orange background; whereas, in Experiment 5, the ratios of S cones and ipRGCs of no-filter/filter condition were 1.50 and 1.44, respectively. It thus seems to support the idea that the blue-light enhancement of DVA only appeared when the activation levels of S cones and ipRGCs differentiated strongly enough.

**Table 2:** Stimulation of cones and ipRGCs of all experiments.

Experiment	Background	L	M	S	ipRGC
Exp. 1	Blue	8.63	6.49	159.89	90.46
	Orange	12.63	3.21	1.93	5.84
	Ratio (blue/orange)	0.68	2.02	82.84	15.49
Exp. 2–4	Blue	6.14	4.53	123.96	66.65
	Orange	8.22	1.91	2.27	3.04
	Ratio (blue/orange)	0.75	2.37	54.61	21.92
Exp. 5	No-filter	23.57	10.13	34.21	33.94
	Filter	21.95	9.82	22.86	23.57
	Celadon	44.93	21.05	61.77	67.85
	Ratio (no-filter/filter)	1.07	1.03	1.50	1.44

## General Discussion

In this study, we found that blue light not only did affect dynamic vision, but also had different effects on distinct types of dynamic vision. In Experiment 1, the blue light enhancement effect was observed on EPA, especially on *y*-axis pursuit, but not on KVA. In Experiment 2, we eliminated the potential masking effect in the KVA task used in Experiment 1, and still found better KVA performance under orange light than under blue light. To further make sure that the differences between EPA and KVA were not due to the task *per se*, we tested DVA, which was similar to KVA but stimuli were presented not only in the fovea but also across bottom and up visual field of vertical motion in Experiment 3. The results showed that DVA could be enhanced under blue light, particularly in downward motion. As the overall DVA performance in the upward condition was significantly better than the downward condition, we hypothesized that the upward motion task was easier than the downward motion task, and that difficulty level mattered. Therefore, in Experiment 4, difficulty level was manipulated by the high and low contrast of stimuli and background, and the results revealed that only in the hard task could DVA be enhanced, confirming our hypothesis. Last but not least, we tested and verified whether the blue light effect on DVA was through ipRGCs activation. Hence, a blue light filter lens was equipped on a goggle to filter out half-level ipRGC activation compared to gray background. The results indicated that DVA enhancement under blue light was not owing to different ipRGCs activations we tested.

The most important finding in this study was that both DVA and EPA could be enhanced under blue light, and that the blue light enhancement effect interacted with difficulty level. Although with a different hypothesis, our findings of DVA enhancement under blue light were consistent with Long and Garvey (1988). Long and Garvey suggested that stimuli on blue background in dark-adapted condition look more vivid than other background. In contrast, we started from the activation of ipRGCs, which had been proven to enhance some cognitive functions and to have relations with motion perception in animal studies. However, neither Long and Garvey nor this study could present strong biological evidence that what mechanisms contribute to the enhanced DVA under blue light. Future studies should modify the current paradigm to examine the neural underpinning of the enhancement for two aspects of dynamic vision (EPA and DVA) under blue light we observed here.

Apart from the blue-light enhancement effect on DVA, we further demonstrated not only the importance of how difficulty level impacted on blue light effect, but also the advantage of perceiving upward motion, compared to downward motion. It is known that when tasks are too easy, performance would reach to the maximum and would gain no benefit no matter what variables are manipulated, so-called the ceiling effect (Salkind, 2010). DVA in upward motion (compared to downward motion, Experiment 2 and 3) and upward motion with black target numbers (compared to gray ones, Experiment 3) was so effortless for participants that they only needed their minimal capacity to complete the task, resulting in no blue light enhancement effect. Future studies could systematically manipulate the difficulty level and test the transition point where blue-light starts to exert its enhancement effect.

The correlation of KVA and EPA in Experiment 1 was only significant under blue light, which indicated that the better performance of EPA, the smaller the impairment of KVA. Brindley et al. (1966) measured the CFFT of blue-, green-, and red-sensitive mechanism, and signified that only blue-sensitive mechanism was impaired. In our KVA task used in Experiment 1, the target numbers appeared at the same position of the fronto-parallel planes and refreshed quickly, in need of the ability of higher temporal resolution like CFFT measured. The lower threshold of KVA under blue light was probably because of the impairment originated from poorer CFFT. Based on this premise, the implication of the correlation may turn out to be that one had better dynamic vision also showed better performance on EPA and less KVA impairment under blue light. Furthermore, KVA without masking effect in Experiment 2 had much better overall performances than in Experiment 1, which may signify that KVA in Experiment 2 was an easier task than in Experiment 1. Also, KVA in Experiment 2 had greater impairment under blue light than that in Experiment 1, indicating a similar pattern of DVA in Experiment 4 that blue-light enhancement could only be observed in hard task. Thus, we could not conclude that blue light enhance EPA and DVA but impair KVA; blue light seemed to play an important role on explicitly enhancing EPA and DVA, and implicitly decreasing the impairment of KVA.

By using blue light filter lens in Experiment 5, we have demonstrated that the blue light enhancement effect on DVA was not caused by different ipRGC activation levels. The next question one might ask is: what mechanism contributes to our findings here? Some may argue that the results might be due to the activation of cones, especially blue-sensitive S cones. Given that blue light impairs temporal and spatial resolution (Cavanagh et al., 1987), if S cones that blue light stimulates most are the main factor for what we observed here, it is still a puzzle why there was improvement of blue light on the DVA and EPA performance in the current study. The low-level features in our KVA and DVA tasks were similar, with black moving numbers as targets on either blue or orange background. The only difference was that the stimuli in KVA were presented in the fovea, which is where the eyes lack the S cones and the ipRGCs. It is thus likely that the blue-light enhancement of EPA and DVA is due to other S-cones related properties like their distribution in the retina.

Could it be possible that S cones have shorter adaptation time than ipRGCs, which leads to the results? Although Wong et al. (2005) found slower light- and dark-adaptation responses in ipRGCs than cones, Do and Yau (2013) found that ipRGCs and cone adaptations are similar in the way that their flash sensitivity adapted in background lights can all be described by the Weber–Fechner law. We therefore consider that the adaptation time course of cones and ipRGCs not a cause for the differences we found here.

In addition to S cones, how did L and M cone activations affect the results? As shown in Table 2, the activations of L and M cones were different – higher L cones activation under orange background and higher M cones under blue background. If L and M cones were the main cause for the results, every experiment in this study should present the same blue-enhancement or blue-impairment. However, background settings in Experiment 2–4 were the same, resulting in better DVA and worse KVA under blue background. Also, both EPA and KVA were measured in Experiment 1, which had the similar result: better EPA but worse KVA. This indicates that L and M cones might not dedicate into the process of the results.

We consider that the different effects among KVA, EPA and DVA might come from post-receptor processes, but not ipRGCs or cones *per se*. It’s well known that there are two visual processing systems: magnocellular system responds fast and is sensitive to luminance contrast, and parvocellular system responds slowly and is sensitive to color (Leventhal et al., 1981; Schiller et al., 1990). Since the magnocellular system processes moving stimuli like black moving digits in the study, and the parvocellular system processes different background colors, they may both contribute to the process of digit discrimination as measured here. The activation of ipRGCs, as well as different types of cones, did not seem to be directly contributed to the different results we obtained.

As for luminance differences, we have tried our best to match the luminance in the blue and orange conditions. It should not be a problem here since this difference is almost neglectable as measured by a spectroradiometer. If luminance difference, albeit small, is an issue, it is also less likely that it would cause a better performance in one condition (EPA and DVA) and worse in the other (KVA). It should not be caused by luminance contrast difference, either, since the target digits were all in black and luminance contrast scales accordingly with luminance.

Due to the luminance setting in our experiments, could rods contribute to the results we obtained here? We think not for the following reasons. (1) In Experiment 5, we have shown that ipRGCs did not seem to contribute to the results. Rods have similar spectral sensitivity function as ipRGs, and thus it is also likely that rods did not contribute to the results. (2) Given that the tasks required discrimination of three digits from 0.91° to 18.43°of visual angle in a rapidly changing situation, high dynamic acuity was needed, which would prevent from rod’s involvements in performing such tasks. (3) Even if rod intrusion is possible, it may be considered as an extension of Purkinje shift, which is also of interest itself. Nonetheless, the luminance level for Purkinje shift is around 0.1 cd/m^2^, which is far below the luminance we had in this study. (4) In fact, rods usually work under 0.01 cd/m^2^ at scotopic level, and under 3 cd/m^2^ for mesopic level, which is also another reason that we consider rod involvement minimal.

Finding biological evidence of spatial suppression under different light conditions could be a possible way to find out how KVA differed from DVA. MT/V5 in human brain is thought to be related to motion perception (Dubner and Zeki, 1971), and this area contains a large amount of direction- and velocity-selective neurons (Albright, 1984). DVA is likely related to the activation of MT/V5 brain regions since it is translational motion on the same plane, especially complex motion like random dots (Antal et al., 2004); KVA is associated with MST areas since it has expansion properties (Tanaka and Saito, 1989). However, neurons in MT/V5 have receptive fields with center–surround antagonism, which may lead to a phenomenon called “spatial suppression,” causing stimuli increased in size becoming hard to perceive (Tadin et al., 2011). As MT signals project to MST (Maunsell and van Essen, 1983), possibly impairing KVA, fMRI is a potential tool to look inside the head. The different brain activations between performing DVA and KVA under blue and orange light could be compared to each other in order to find the decisive proofs.

Studies on dynamic vision usually aim at diseases, measurement for diagnosis, or training for athletes, and basic research from psychological and biological fields are severely lacking. Although dynamic vision could not be clearly separated from other associated abilities, it still has its own unique properties. In real life, we usually see things in 3-D space and are more aware of moving objects, but studies to date focus more on 2-D static vision. We have shown, in this first study demonstrating the relationship between different components of dynamic vision and blue light, that DVA can be enhanced under blue light with hard but not easy task, indicating that blue light can increase the threshold of dynamic visual discrimination when needed. Future studies can help clarifying the pros and cons of different approaches of measuring dynamic vision, and to investigate under what other circumstances can dynamic vision be enhanced. Hopefully through a systematic approach, better understanding of dynamic vision can be achieved and methods can be developed to improve dynamic vision on brain lesion patients, athletics demanding on perceiving fast-moving targets, or even video game players.

## Author Contributions

H-WC and S-LY designed this study and wrote the manuscript. H-WC conducted the experiments, collected the data and analyzed the data. S-LY supervised the whole work.

## Conflict of Interest Statement

The authors declare that the research was conducted in the absence of any commercial or financial relationships that could be construed as a potential conflict of interest.
